# Food Insecurity Is Common in Patients with Inflammatory Bowel Disease and Is Associated with Increased Ultra-Processed Food Intake

**DOI:** 10.3390/nu16213736

**Published:** 2024-10-31

**Authors:** Stephanie Lauren Gold, David Kohler, Hannah Freid, Natasha Haskey, Maitreyi Raman

**Affiliations:** 1 The Henry D. Janowitz Division of Gastroenterology, Icahn School of Medicine at Mount Sinai, New York, NY 10029, USA; stephanie.gold@mountsinai.org; 2 Department of Pediatrics, Icahn School of Medicine at Mount Sinai, New York, NY 10029, USA; 3 Icahn School of Medicine at Mount Sinai Hospital, New York, NY 10029, USA; 4 Department of Biology, University of British Columbia—Okanagan, Kelowna, BC V1V 1V7, Canada; natasha.haskey@ubc.ca; 5 Department of Medicine, Division of Gastroenterology and Hepatology, University of Calgary, Calgary, AB T2N 1N4, Canada

**Keywords:** food insecurity, inflammatory bowel disease, NOVA score, diet quality, processed foods

## Abstract

Background/Objectives: Food insecurity (FI) is defined as the lack of consistent access to enough food for an active and healthy life. FI affects over 30 million Americans and is associated with poor clinical outcomes and impaired quality of life and drives significant health inequities. Despite the rising prevalence of FI and the federal focus on improving access to healthy food, there is a paucity of research on FI in patients with inflammatory bowel disease (IBD). Therefore, the goal of this study was to define FI in a cohort of IBD patients and determine whether FI was associated with changes in dietary patterns, including specifically an increase in ultra-processed food (UPF) consumption in this high-risk patient population. Methods: This was a single-center, retrospective cohort study of patients with a diagnosis of IBD who were 18 years of age or older and who were seen in a nutrition focused clinic. Patients were screened for FI using the Hunger Vital Sign™, a 2-question validated FI screening tool and underwent a 24-h dietary recall. The degree of food processing was assessed using the NOVA Food Classification System. Results: Among 128 patients with IBD, we observed that FI is increasingly prevalent, with 45% of patients reporting difficulty with sufficient grocery access at least “sometimes” in the last 12 months and 10% reporting decreased food access “often” in the prior year. In addition, the patients at high-risk for FI were significantly more likely to eat NOVA 4 UPFs (54% vs. 27%, *p* = 0.001) and were significantly less likely to eat NOVA 1 unprocessed foods (32% vs. 61%, *p* = 0.001) as compared to those not at risk for FI. Finally, only a small percentage of those at highest risk for FI were enrolled in a federal food assistance program for grocery support. Conclusions: The prevalence of FI is increasing in patients with IBD and is associated with reduced dietary quality.

## 1. Introduction

Food insecurity (FI), or the lack of consistent access to enough food for an active and healthy life, is an important social determinant of health [1]. FI affects over 30 million Americans and is associated with poor clinical outcomes and impaired quality of life and drives significant health inequities. With the current economic situation and rising inflation rates, the cost of food has increased exponentially, leading to fewer donations to food banks and higher rates of FI, deepening health disparities [2]. Despite the rising prevalence of FI and the federal focus on improving access to healthy food, there is a paucity of research on FI in patients with inflammatory bowel disease (IBD). There is a single study published on FI and IBD that estimated the prevalence to be 12.5% [3]. However, this study utilized data from 2015 and therefore did not consider the impact of the COVID-19 pandemic and subsequent economic changes. Despite the lack of current data on FI in IBD, studies from the general population have shown that with rising food costs, individuals compensate by reducing the nutritional quality of the food they eat and opt for lower cost alternatives, including fewer fruits and vegetables and more processed foods [4].

In the United States (US), the term ultra-processed foods (UPFs) first emerged in the 1980s, describing popular convenience foods with emulsifiers, thickeners, artificial colors, and sweeteners [5]. However, food processing accelerated many years prior, during World War II, as the ability to package and distribute food to prevent starvation was considered a lifesaving advance. The transition from small local farming to industrialized food production using additives and preservatives led to the development of convenience foods and ready-to-eat meals that could be distributed worldwide. While this advance saved thousands of lives during the war, once the crisis had ended, the consumption of these processed and packaged foods continued to increase. A recent systematic review including 99 studies across 20 countries reported that ultra-processed food (UPF) intake varied significantly worldwide, with the highest rates in the US and United Kingdom (>50% of the diet) [6].

Over the last decades, the potential harms associated with UPFs have been highlighted across medical disciplines, with public health initiatives trying to label, tax, and even ban these foods. Studies have shown that UPFs are associated with increased all-cause mortality, cardiometabolic disease, psychiatric disorders, and other chronic illnesses [7]. Recent studies have suggested that diets rich in UPFs are associated with increased gastrointestinal illness, including increased rates of IBD [7]. Early evidence suggests that consumption of food additives including thickeners, artificial sweeteners, and colors may lead to fecal dysbiosis, changes in the intestinal mucous layer, as well as the immune cells in the lamina propria, inhibiting anti-inflammatory short chain fatty acid production and leading to intestinal inflammation [8]. In fact, in a large prospective cohort study including over 10,000 individuals, a higher intake of UPFs (including processed meats, soft drinks, snacks, and artificial sweeteners) was associated with an increased risk of developing IBD [9,10]. A subsequent study confirmed the increased risk of developing Crohn’s disease (CD) in those with higher UPF intake; however, it did not see an association between UPFs and the development of ulcerative colitis (UC) [9,11]. These findings have been replicated in subsequent cohort studies [7,12,13].

Given the impact of UPFs on the microbiome and intestinal inflammation, understanding how consumption of these foods impacts IBD outcomes is of significant interest. Furthermore, the connection between FI, UPFs, and the pathogenesis of IBD as well as the disease course remains unknown. Therefore, the goal of this study was to define FI in a cohort of IBD patients and determine whether FI was associated with an increase in UPF consumption in this high-risk patient population.

## 2. Materials and Methods

### 2.1. Patient Selection

This was a single-center, retrospective cohort study of patients with a diagnosis of IBD who were 18 years of age or older. Patients in this study were part of a larger, prospective cohort of patients seen in a multi-disciplinary nutrition-focused clinic in our quaternary care IBD center located in a large, diverse, metropolitan city within the US from 2022 to 2023. This clinic includes patients who were previously identified as high risk for malnutrition (using the Malnutrition Universal Screening Tool) and, therefore, referred for comprehensive nutrition care. Patient demographics and disease characteristics, including IBD type, medication history, disease activity, surgical history, and supplement use, were collected at the initial visit from structured questionnaires and medical record review. All patients were evaluated for malnutrition utilizing the Global Leadership Initiative on Malnutrition (GLIM) Diagnostic Criteria. Inclusion criteria included any patient over the age of 18 years with a diagnosis of IBD who was seen for a standard of care malnutrition visit. Exclusion criteria included any patient under the age of 18 years, those without a diagnosis of IBD, and those who did not complete the standard of care FI screener.

### 2.2. Food Insecurity Screening

As part of the pre clinic visit questionnaire, patients were screened for FI using the Hunger Vital Sign™, a 2-question validated FI screening tool [14] (Supplement S1). This tool was developed in 2010 based on the US Household Food Security Survey module to identify those individuals or households at risk of FI [14]. Patients were identified as being at risk of food insecurity if they responded to either of the two statements included in the screening tool with “often true” and/or “sometimes true”. Those not at risk of FI were defined as those who responded “never true” to both screening questions. While this tool has not been studied in an IBD cohort, it has been validated in the general adult US population [15]. Finally, patients at risk of FI were asked about enrollment in any federal assistance program, including the Supplemental Nutrition Assistance Program (SNAP). Previously known as the Food Stamp Program, SNAP is a federal assistance program that provides a monthly stipend to purchase nutritious groceries for those with low and no income [16].

### 2.3. Dietary Assessment

All patients in this study underwent a 24-h dietary recall during the clinic visit. This recall was performed by a gastroenterologist with specialized nutrition training. Dietary recalls included breakfast, lunch, dinner, snacks, and drinks consumed on the day prior to the clinic visit. Any patient who was not able to provide this information was excluded from the study. Patients on total parenteral nutrition (TPN) or partial parenteral nutrition (PPN) were also excluded from the study. The degree of food processing was assessed using the NOVA Food Classification System. This previously validated tool includes four categories: group 1: unprocessed or minimally processed foods, group 2: processed culinary ingredients, group 3: processed foods, and group 4: UPFs. This classification system has been widely used in the literature, both among IBD patients and across medical disciplines as well as in population level studies [17,18,19,20,21]. Utilizing a previously published technique to assess processing in the diet with the NOVA Food Classification System, each food item consumed (including solid food and drinks) was assigned a NOVA score (1–4) and subsequently weighted to determine the percentage of food items in each category consumed per day [22]. The quantification of NOVA groupings was conducted by the number of food items reported in the 24 h recall and not by the percentage of calories or weight of the foods detailed. Of note, oral nutrition supplements were excluded from the analysis, given the controversy of whether these should be considered medications vs. UPFs etc. [23]. In addition, enrollment in the Supplemental Nutrition Assistance Program (SNAP) was recorded.

### 2.4. Statistical Analysis

Descriptive statistics were used to summarize the data including Student T tests for continuous variables and Fisher exact tests for categorical (binary) variables. Univariate analysis was used to test associations using SAS Statistical Software V.9.3 (Cary, NC, USA).

### 2.5. Ethical Considerations

The study was conducted according to the ethical principles of the Declaration of Helsinki. This study was approved by the Institutional Review Board at the Icahn School of Medicine at Mount Sinai.

## 3. Results

In total, 128 adult patients with a diagnosis of IBD were included for analysis. In this study, 61 (49%) patients were female with an average age of 39.8 years. In addition, 23 (18%) self-reported as Hispanic or Latino and 89 (70%) had Crohn’s disease. The average BMI in the study was 20.5 kg/m^2^. In total, 36% of patients were diagnosed with malnutrition based on the GLIM criteria (definition in Supplement S2) Full demographic data and disease characteristics are summarized in Table 1.

**Figure 1:**
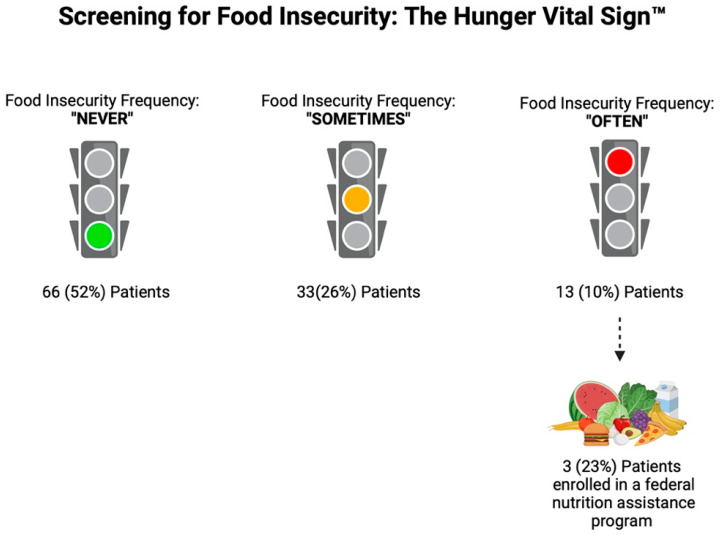
Screening for Food Insecurity using the Hunger Vital Sign ^TM^. Figure created with biorender.com.

Analyzing the FI questionnaires, 4 (3%) patients responded “often” to both of the FI questions, 9 (7%) patients responded “often” to one of the questions, 33 (26%) patients responded “sometimes” to one of the questions, 16 (12.5%) patients responded “sometimes” to both questions, and 66 (52%) patients responded never to both questions. Overall, based on the Hunger Vital Sign™ recommended scoring, 62 (48%) patients were identified as “at risk for FI”, with 13 (10%) patients at highest risk (those that responded “often true” to one or both of the screening questions). Of these 13 patients who responded “often true” to one or both of the screening questions, only 3 (23%) were enrolled in the SNAP program (Figure 1).

**Figure 2:**
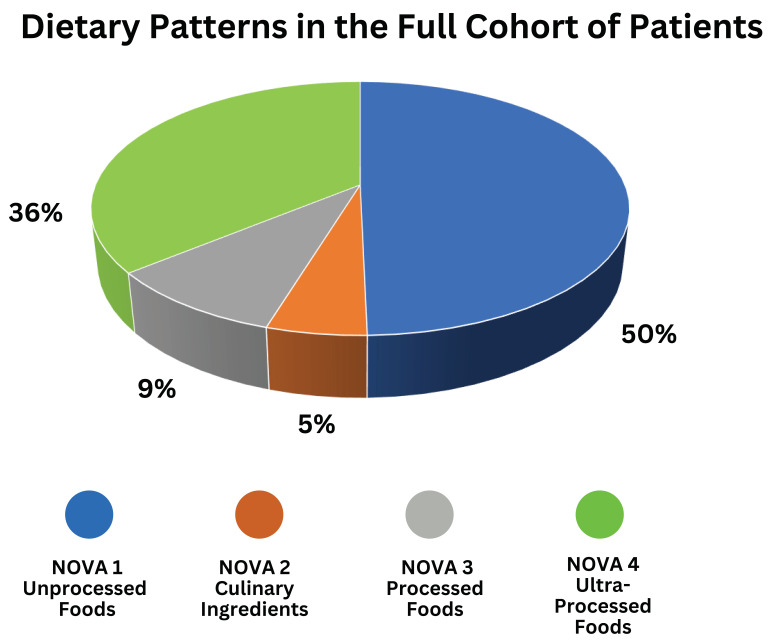
Classification of dietary patterns (degree of food processing) using the NOVA classification system in the full study cohort.

All 128 patients in this study underwent a dietary assessment with a 24-h recall. In the full cohort, patients reported an average of 11.3 foods consumed daily, with 50.1% of foods from the NOVA 1 group, 5.1% from the NOVA 2 group, 9.4% from the NOVA 3 group, and 36% from the NOVA 4 group (Figure 2). Twenty (15.6%) patients reported drinking oral nutrition supplements in the 24-h recall.

**Figure 3:**
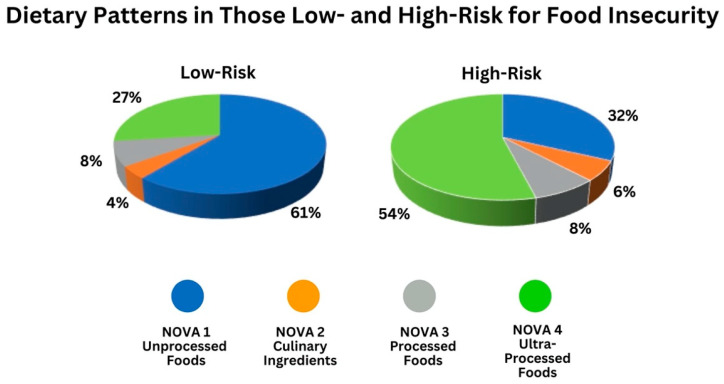
Classification of dietary patterns (degree of food processing) using the NOVA classification system in patients at high risk for food insecurity and those at low risk for food insecurity.

Comparing those patients at high risk for food insecurity with those not at risk for food insecurity, there was no significant difference in the number of food items reported in the 24-h recall (11.1 vs. 11.2). In this cohort, the patients at high-risk for FI were significantly more likely to eat NOVA 4 UPFs (54% vs. 27%, *p* = 0.001) and were significantly less likely to eat NOVA 1 unprocessed foods (32% vs. 61%, *p* = 0.001) as compared to those not at risk for FI (Figure 3, Table 2). There was no difference in the percentages of NOVA 2 or NOVA 3 foods consumed between those at high risk for FI and those not at risk of FI (NOVA 2: 5.8% vs. 4.7%%, NOVA 3: 8.2% vs. 8.7%). Those at high risk for FI were more likely to be drinking oral nutrition support shakes compared to those not high risk for FI, although this difference did not reach statistical significance (23% vs. 13.8%, *p* = 0.416). Finally, patients who were identified as at risk of FI were significantly more likely to be malnourished, as defined by GLIM (63% vs. 12%, *p* < 0.001).

## 4. Discussion

In this single-center cohort study, we observed that FI is increasingly prevalent among a diverse IBD population seen in a nutrition-focused clinic, with 36% of patients diagnosed with malnutrition at the clinic visit using the GLIM criteria. In this cohort, 45% of patients reported difficulty with sufficient grocery access at least “sometimes” in the last 12 months and 10% reporting decreased food access “often” in the prior year. In addition, those who were at highest risk for FI consumed significantly more UPF and significantly fewer unprocessed foods compared to those not at risk for FI. Finally, only a small percentage of those at highest risk for FI were enrolled in a federal food assistance program for grocery support.

To date, there is a single study evaluating the prevalence and impact of FI in patients with IBD. This study was focused on financial toxicity associated with healthcare use and indirectly assessed FI as one of the social determinants of health [3]. Utilizing data from the National Health Interview Survey 2015, the study estimated that 1 in every 8 patients (12.5%) with IBD was identified as at risk of FI [3]. The study concluded that FI was associated with increased financial hardship from medical bills (odds ratio (OR) 3.31, 95% confidence interval (CI) 1.48–7.39) and medication nonadherence due to cost (OR 8.07, 95% CI 3.16–20.6) [3]. While this study utilized a large dataset to estimate the prevalence of FI in patients with IBD, the survey was from 2015 and, therefore, does not take into account the impact of the COVID-19 pandemic and the current economic conditions with rising inflation and rapidly increasing food prices [24]. Utilizing data from 2007–2016 NHANES, among 9190 adults aged 20–65 years, more severe FI was associated with increased consumption of UPFs, as defined by the NOVA classification (*p* = 0.003) [25]. While FI certainly leads to changes in dietary patterns, it is also intimately related to healthcare utilization; a recent survey study demonstrated that 21% of those who identified as FI delayed medical care or necessary procedures, while an additional 13% did not purchase prescribed medications; these rates are even higher among patients with chronic medical conditions (23% delayed or skipped medical care and 16% did not purchase prescription medications) in order to put meals on the table [26]. Given the rising prevalence of FI identified in our study and the impact not only on food choices but also on healthcare utilization, compliance, and quality of life, it is crucial that patients with IBD be screened for FI and those at high risk should be offered resources, including federal assistance programs, local food pantries, and other available community programs.

**Figure 4:**
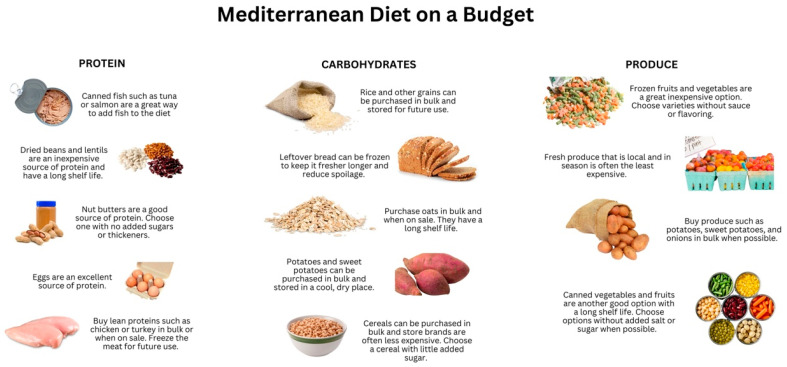
Food recommendations for patients following a Mediterranean style diet on a budget, broken down by protein sources, carbohydrates, as well as fruits and vegetables.

Patients with IBD are generally encouraged to follow a Mediterranean style diet with plentiful fruits and vegetables, whole grains, lean protein sources, and olive oil while limiting processed foods, food additives, and red meat. For patients who are at risk of FI, this can be quite challenging. In this study, those at the highest risk of FI had the lowest intake of NOVA group 1 foods, which includes fruits, vegetables, lean meats, legumes, fish, eggs, and unprocessed whole grains (the main components of a Mediterranean diet). Once patients are identified as food insecure, providers can counsel patients on how to follow a Mediterranean style diet while on a tight budget. These recommendations include consumption of canned fruits and vegetables (packed in water without sugar or salt), canned beans, oats, no sugar added peanut butter, canned fish such as tuna or salmon, and purchasing potatoes and frozen fruit or vegetables in bulk (Figure 4). Given the limited access to dedicated IBD dietitians globally, it is crucial to establish standardized recommendations for patients with IBD experiencing FI, such as the ones provided in Figure 4, that can be modified for those with active disease in comparison to those in remission and that can be delivered by any provider.

While the NOVA Food Classification System is very commonly used in dietary literature and has been utilized by federal agencies and the United Nations, there are some important limitations to this tool. The majority of foods listed in the NOVA 1 group are universally accepted as unprocessed, including fruits and vegetables, whole grains such as rice or quinoa, fresh meats, eggs, lentils, beans, unsalted nuts, herbs and spices, water, coffee, and tea. Similarly, the majority of the foods listed in the NOVA 4 group are universally accepted as UPFs, including packaged snacks, cookies, hot dogs and sausages, instant meals, soft drinks, sports drinks, preprepared fish or poultry nuggets, and sliced deli meats. However, there are many food items that, while ultra-processed, can be considered an integral part of a healthy diet, especially in IBD patients who are struggling with food access. For example, fortified, unsweetened breakfast cereals are a good, low-cost option. These cereals are generally well tolerated in patients with IBD regardless of disease activity and, in those with stricturing CD, can provide the desired textures from food without increasing the risk of obstruction. In addition, oral nutrition supplements, while considered part of the NOVA 4 UPF group, have been shown to induce clinical and endoscopic remission in up to 70% of patients with CD on exclusive enteral nutrition [27,28]. Given the data suggesting that food additives such as emulsifiers, thickeners, artificial sweeteners, dyes, and preservatives may contribute to dysbiosis and intestinal inflammation, perhaps this should be the focus of dietary restriction and not location of production or degree of processing (unrelated to additives). For example, as per the NOVA classification, if a bread is made with whole ingredients and no food additives at home, this is a NOVA 1 food; however, if the same bread is made in a commercial factory, this is considered a NOVA 4 food. Further studies will need to elucidate whether it is the processing of food, as NOVA suggests, or the ingredients in that food that have the biggest impact on clinical outcomes in patients with IBD.

In this study, patients who were identified as FI were more likely to be malnourished, as defined by the GLIM criteria, compared to those who were not FI. This association has been similarly described across medical disciplines [29]. Global epidemiologic studies have demonstrated that FI is one of the major causes of malnutrition worldwide and is associated with a myriad of poor health outcomes, including, but not limited to, hypertension, diabetes, depression, and anxiety [30]. In patients with IBD, malnutrition is associated with disease specific complications including impaired response to biologics, more frequent disease flares, and increased surgical complications, as well as decreased quality of life [31]. Given that FI is a significant risk factor for reduced dietary quality and malnutrition, it is imperative to screen patients with IBD for FI at the time of diagnosis and regularly thereafter as part of a comprehensive nutrition screening/assessment.

This study has several potential limitations. First, while the dietary assessment was conducted prospectively, the NOVA classification was completed retrospectively and some food items had to be excluded due to insufficient information. Although rare, examples include a patient reporting consumption of peanut butter with no additional details about brand (use of salt, sugar, or additives) or food items consumed in a restaurant (as the ingredients were not known). Moreover, the retrospective nature of this dietary analysis could have introduced bias due to the missing data on certain food items. Second, the FI screening was conducted as part of a multi-disciplinary nutrition and IBD clinic; this may have naturally identified those who had questions about food resources and therefore the prevalence of FI in this cohort may be higher than that in the general IBD clinic. Further studies in the general IBD patient population will help clarify this. Moreover, utilization of a standardized dietary recall tool, such as the ASA24, instead of a 24-h recall would have allowed detailed information on portion sizes to be obtained and the percentage of foods in each NOVA category per total calories or total grams of food consumed each day could have been calculated. Instead of only assessing food items, this would provide significantly more information about the overall amount of food consumed; if patients were to consume a large portion of a NOVA 1 food and one bite of a NOVA 4 food, this would be lost without calculating the foods as a percentage of total weight or calories consumed. Finally, this study was conducted in a large metropolitan US city and therefore the results may not be applicable to other communities, both nationally and internationally, where food access and dietary options may vary.

## 5. Conclusions 

In summary, this is the first study to assess the current prevalence of FI and its associated dietary changes in patients with IBD, including the significant impact of the COVID-19 pandemic and the subsequent economic changes. With the diverse, specialized cohort of patients with IBD who were referred for malnutrition assessment included in this study, there were a significant number of patients at risk for FI, demonstrating the immense importance of screening patients using the 2-question Hunger Vital Sign™ tool. Future studies are needed to assess the prevalence of FI in other national and international communities, including both urban and rural locations. Given the stigma associated with FI and use of federal assistance programs and/or local food pantries, many patients will not mention this information unless specifically asked by a provider. These objective, validated questions can help easily and rapidly screen patients for FI and provide those at risk with the resources they need to eat a healthy, Mediterranean style diet and ultimately improve disease control.

## Figures and Tables

**Table 1:** Cohort Characteristics.

Characteristics		Study Cohort (*n* = 128)
Age (mean, years, [range])		39.6 (19–77)
Biologic Sex, Female n (%)		61 (47)
Ethnicity	Hispanic or Latino, n (%)	23 (18)
	Not Hispanic or Latino, n (%)	106 (82)
Race	White, n (%)	88 (68)
	African American, n (%)	12 (9)
	Asian, n (%)	6 (4)
	Native American, n (%)	0 (0)
	Other, n (%)	15 (12)
	Not provided, n (%)	8 (6)
Type of IBD	Crohn’s Disease, n (%)	89 (70)
	Ulcerative Colitis, n (%)	36 (28)
BMI (kg/m^2^)	<18.5	55 (43)
	18.5–24.9	55 (43)
	25–29.9	12 (9)
	30–39.9	6 (5)
	≥40	0 (0)
Disease Phenotype (Montreal Classification)	CD: L1 Ileal, n (%)	24 (27)
	CD: L2 Colonic, n (%)	11 (13)
	CD: L3 Ileocolonic, n (%)	54 (60)
	CD: B1 Non Stricturing, n (%)	45 (50)
	CD: B2 Stricturing, n (%)	33 (37)
	CD: B3 Penetrating, n (%)	11 (12)
	CD: Perianal Disease, n (%)	23 (26)
	UC: E1: Proctitis, n (%)	2 (5)
	UC: E2: Left Sided, n (%)	9 (25)
	UC: E3: Pancolitis, n (%)	25 (70)
Prior IBD related surgery		44 (34)
IBD Activity	HBI, mean (range)	4.7 (0–13)
	pMayo, mean (range)	2.5 (0–7)
Malnourished (GLIM)		47 (36)

IBD: Inflammatory bowel disease, HBI: Harvey Bradshaw Index, pMayo: partial Mayo Score, GLIM: Global Leadership Initiative on Malnutrition.

**Table 2:** (**a**,**b**): Percentage of Food Items in Each NOVA Category and Food Access.

(**a**)				
**Food Access**	**Percentage of Food Items**			
	**NOVA 1**	**NOVA 2**	**NOVA 3**	**NOVA4**
Not Food Insecure	61.4%	4.7%	8.7%	27.1%
Food Insecure	38.5%	5.6%	10.0%	45.8%
(**b**)				
**Food Insecurity**	**Percentage of Food Items**			
	**NOVA 1**	**NOVA 2**	**NOVA 3**	**NOVA4**
Food Insecurity Survey: Never	61.4%	4.7%	8.7%	27.1%
Food Insecurity Survey: Sometimes	40.2%	5.6%	10.3%	43.8%
Food Insecurity Survey: Always	32.2%	5.9%	8.2%	32.2%

## Data Availability

The original contributions presented in the study are included in the article, further inquiries can be directed to the corresponding author.

## Supplementary Materials

The following supporting information can be downloaded at: https://www.mdpi.com/article/10.3390/nu16213736/s1, Supplement S1: Screening for Food Insecurity: The Hunger Vital Sign™; Supplement S2: Global Leadership Initiative on Malnutrition (GLIM) Diagnostic Criteria.
