# Supplementary material for: Food Insecurity Is Common in Patients with Inflammatory Bowel Disease and Is Associated with Increased Ultra-Processed Food Intake
Source: Nutrients. 2024 Oct 31;16(21):3736. doi: 10.3390/nu16213736 (PMC11547406; doi:10.3390/nu16213736)
Supplement: Supplementary file 1 [file nutrients-16-03736-s001.zip › nutrients-3252778-supplementary.pdf]

Supplement S1: Screening for Food Insecurity: The Hunger Vital Sign™

Question 1: Within the past 12 months, I worried whether my food would run out before I got money to buy more:

- Often True
- Sometimes True
- Never True

Question 2: Within the past 12 months, the food I bought just didn't last and I didn't have enough money to get more.

- Often True
- Sometimes True
- Never True

Patients at risk for food insecurity: Answered "Sometimes true" or "Often true" to either question above.
Patients not at risk for food insecurity: Answered "Never true" for both questions

Supplement S2:

Global Leadership Initiative on Malnutrition (GLIM) Diagnostic Criteria

	**Phenotypic Criteria**			**Etiologic Criteria**	
	**Unintentional Weight Loss**	**Body mass Index (kg/m^2)**	**Low Muscle Mass**	**Reduced Food Intake or Assimilation**	**Inflammation**
Traditional Definition	>5% in the last 6 months or >10% beyond 6 months	<20 if 70 years OR <22 if >70 years	Reduced muscle mass	-≤50% of energy requirement consumed in >1 week<br>-Any reduction in oral intake for >2 weeks<br>-Any chronic GI condition that adversely impacts food assimilation	-Acute disease/injury<br>-Chronic disease/injury
IBD Specific Modifications	No change	No change	No change	- ≤50% of energy requirement consumed in >1 week<br>-Any reduction in oral intake for >2 weeks (defined as a reduction from baseline (including restriction of major food groups)<br>-Any chronic GI condition that adversely impacts food assimilation (Defined as any prior IBD related luminal surgery including presence of an ileostomy, small bowel resection, colectomy, IPAA, ICR)	Acute disease/injury<br>-Chronic disease/injury (Defined as an abnormally increased serum biomarker [C-reactive protein or erythrocyte sedimentation rate] OR elevated calprotectin OR if biomarkers unavailable, active disease based on clinical disease assessment metrics (HBI/pMayo).

IPAA: ileal pouch-anal anastomosis
HBI: Harvey Bradshaw Index
pMayo: Partial Mayo Score
